# Where to Vaccinate

**Published:** 1902-05-10

**Authors:** 


### WHERE TO VACCINATE.

Dr. Wilson Parry[1] raises the interesting question what region of the body surface should a medical man choose on which to be vaccinated, supposing for any cause he wishes to perform that operation on himself? The site he selected in his own case was the space over the lower part of the sternum, between the breasts, the centre of the area being a point on the horizontal nipple line midway between the two nipples. This spot is convenient for self operation and also for self inspection; secondly he hoped that, supposing some inflammation were to take place it would not interfere with the movement of his limbs; thirdly, the spot selected is well sheltered, lying as it does between the slightly projecting male breasts; fourthly, he thought that there would be less likelihood of glandular implication, in consequence of the absorbed products being distributed among two sets of absorbents. In all these points the result in his own case answered all his expectations. Evidently all his reasons, except the first, would apply to the revaccination of other active men besides medical practitioners.

[1] Lancet, April 19.

